# Comparative Studies of Recirculatory Microbial Desalination Cell–Microbial Electrolysis Cell Coupled Systems

**DOI:** 10.3390/membranes11090661

**Published:** 2021-08-27

**Authors:** Desmond Ato Koomson, Jingyu Huang, Guang Li, Nicholas Miwornunyuie, David Ewusi-Mensah, Williams Kweku Darkwah, Prince Atta Opoku

**Affiliations:** 1 Ministry of Education Key Laboratory of Integrated Regulation and Resource Development on Shallow Lakes, College of Environmental Engineering, Hohai University, No. 1 Xikang Road, Nanjing 210098, China; descy06@hhu.edu.cn (D.A.K.); nickchristos7@gmail.com (N.M.); ewusimensah.david@gmail.com (D.E.-M.); williams.darkwah@stu.ucc.edu.gh (W.K.D.); megee138@outlook.com (P.A.O.); 2 Key Laboratory of Songliao Aquatic Environment, Ministry of Education, Jilin Jianzhu University, Changchun 130118, China; liguang1908@163.com

**Keywords:** recirculatory MDC–MEC coupled system, heavy metals removal, power source, ammonium ions, single-chambered MEC, dual-chambered MEC

## Abstract

The recirculatory microbial desalination cell–microbial electrolysis cell (MDC–MEC) coupled system is a novel technology that generates power, treats wastewater, and supports desalination through eco-friendly processes. This study focuses on the simultaneous efficient removal of Fe^2+^ and Pb^2+^ in the MEC and ammonium ions in the MDC. It also evaluates the performances of dual-chambered MEC (DCMEC) and single-chambered MEC (SCMEC), coupled with MDC with Ferricyanide as catholyte (MDCF) in heavy metals (Pb^2+^ and Fe^2+^) removal, in addition to the production of voltage, current, and power within a 48-h cycle. The SCMEC has a higher Pb^2+^ (74.61%) and Fe^2+^ (85.05%) removal efficiency during the 48-h cycle than the DCMEC due to the simultaneous use of microbial biosorption and the cathodic reduction potential. The DCMEC had a higher current density of 753.62 mAm^−2^ than that of SCMEC, i.e., 463.77 mAm^−2^, which influences higher desalination in the MDCF than in the SCMEC within the 48-h cycle. The MDCF produces a higher voltage (627 mV) than Control 1, MDC (505 mV), as a power source to the two MECs. Stable electrolytes’ pH and conductivities provide a conducive operation of the coupled system. This study lays a solid background for the type of MDC–MEC coupled systems needed for industrial scale-up.

## 1. Introduction

Portable drinking water is essential for the survival of most living things, especially humans. Due to deforestation, overconsumption, heavy metal pollution, chemical pollution, and the impact of global warming, the amount of potable freshwater is gradually reducing [1,2,3,4]. Water bodies polluted with heavy metals and ammonia from industries have become a global concern [1,2,3,4,5]. Various techniques for removing heavy metals and ammonia from wastewater have been researched in recent years [6,7,8]. Some of these techniques include coagulation-flocculation, ion exchange, adsorption, flotation, membrane filtration, chemical precipitation, and electrochemical and bio-electrochemical methods including microbial electrolysis cells (MEC) [6,7,8]. Other conventional methods of ammonium ions removal include chemical immobilization, physical process, and adaptation of microbes, while new technologies include hollow fiber membranes, ultrasonication, microwaves, and microbial electrochemical cells [9,10,11,12]. Ammonia in the form of ammonium chloride (NH_4_Cl) can be desalinated from wastewater. Several technologies including reverse osmosis, electrodialysis, bioremediation, and bio-electrochemical systems (BES), such as microbial fuel cells (MFC) and microbial desalination cells (MDC), have been used to treat and desalinate wastewater or brackish water for reuse [13,14,15,16,17,18,19,20,21]. These systems, when operated independently, have several limitations that impede their performance. Some of these technologies, such as water electrolysis, depend on an external power source. which greatly affects its energy consumption and cost of heavy metals removal or hydrogen gas production.

**Figure 1:**
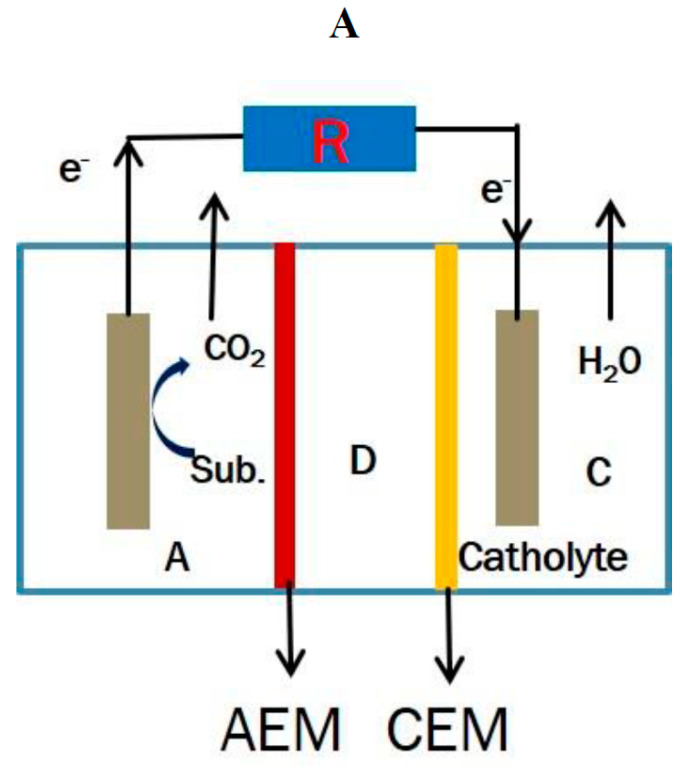

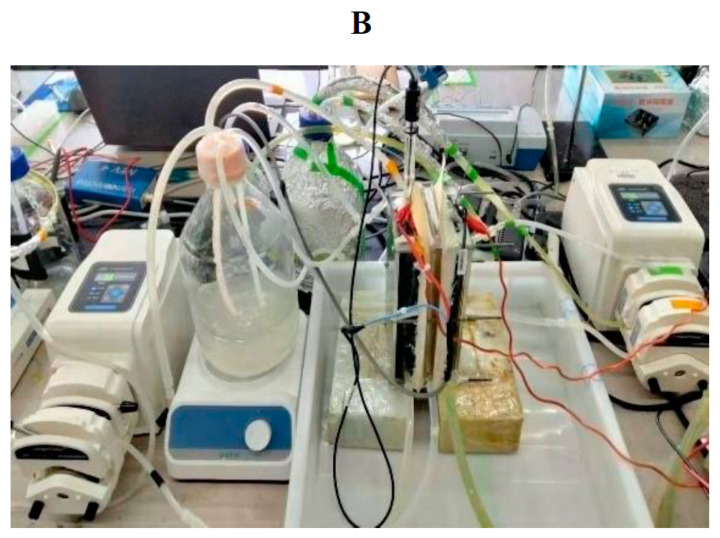
(**A**) Schematic diagram of a conventional MDC. Note: A, anode chamber; D, desalination chamber; C, cathode chamber; and R, external resistance. (**B**) Experimental diagram of a conventional MDC.

**Figure 2:**
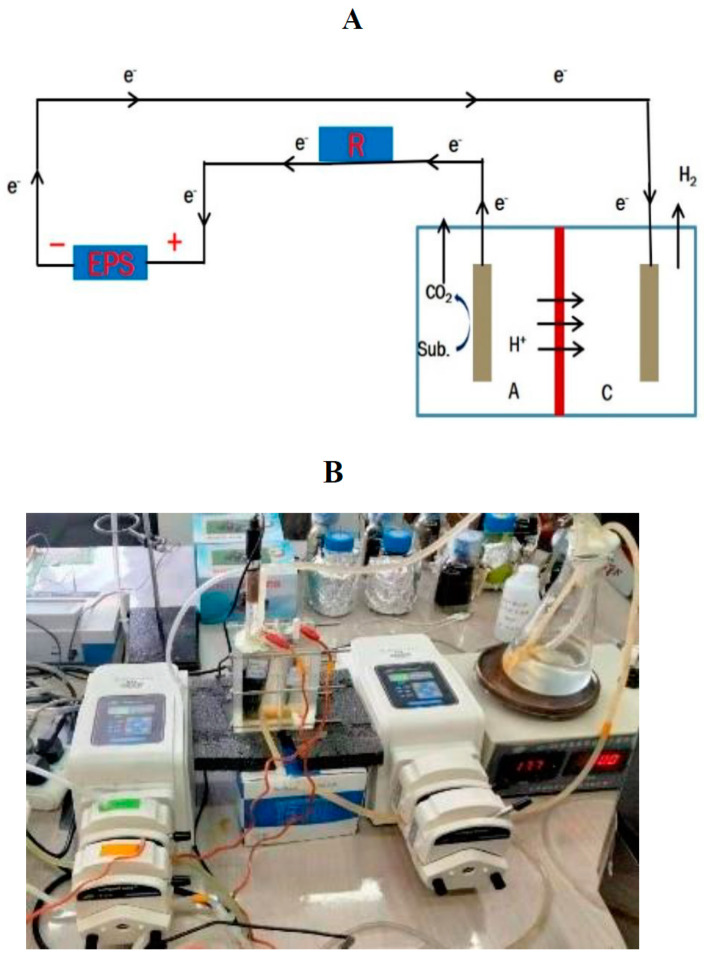
(**A**) Schematic diagram of the dual-chambered MEC. Note: A, anode chamber; D, desalination chamber; C, cathode chamber; R, resistance; and EPS, external power supply. (**B**) Experimental diagram of the dual-chambered MEC.

The significant benefits of treating wastewater imply the use of a coupled BES that operates efficiently and has negative energy consumption [22,23,24]. Bio-electrochemical coupled systems (BECS) are very important and efficient in several processes including wastewater treatment, heavy metal removal, desalination, and chemical production [22,24,25,26]. In recent years, several BECS such as the microbial desalination cell–microbial electrolysis cell (MDC–MEC), microbial fuel cell–microbial electrolysis cell (MFC–MEC), and constructed wetlands–microbial fuel cell (CW–MFC) have been developed [22,24,25,26]. BECS enhance the performance of an individual bio-electrochemical system (BES). The MDC–MEC coupled system is a typical BECS that is a very self-sustaining system that improves the operations and performances of the individual MDC and MEC systems. The MDC–MEC coupled system is comprised of a microbial desalination cell (MDC) that powers a microbial electrolysis cell (MEC) for processes such as gas and chemical production and heavy metal reduction. The MDC is a three (3) chambered system that treats wastewater, desalinates saline water, and generates power as shown in Figure 1. MEC is usually powered by an external power supply as seen in Figure 2. Exoelectrogens in the anode chambers of these bioreactors produce electrons by oxidizing carbon sources (including glucose and acetate) as described in Equations (1) and (2). The production of electrons by exoelectrogens is a key component in the MDC–MEC coupled system for the current generation, desalination, and heavy metal reduction [17,27].

C_2_H_4_O_2_ + 2H_2_O → 2CO_2_ + 8e^−^ + 8H^+^ (1)

C_6_H_12_O_6_ + 6H_2_O → 6CO_2_ + 24H^+^ + 24e^−^ (2)

Several heavy metals have been removed from wastewater using MEC [22,28,29,30] but the performance of the two major types of MEC in this process (DCMEC and SCMEC) have not been evaluated. In this study, lead (Pb) and iron (Fe) were reduced in the two MEC systems. The reduction potential (E°) of Pb and Fe are −0.13 V and −0.45 V as shown in Equations (3) and (4). For Pb and Fe to be reduced at the cathode of the MEC, the MDC needs to reduce the cathode potential of the MEC to below −0.5 V experimentally. Desalination is a key component of the MDC-MEC coupled system. The current generation is influenced by desalination in the middle chamber of the MDC. High saline water (seawater or brackish water) or wastewater with high salinity can be desalinated in the MDC. In this study, NH_4_Cl was desalinated in the MDC. The current generated through desalination and substrate oxidation is supplied to the MEC, hence, maintaining a self-sustaining system. This study will help in the scaling-up of the MDC–MEC coupled system.

Reactions at the cathode of the MEC:

Fe^2+^_(aq)_ + 2 e^−^ → Fe_(s)_    E° = −0.45 V vs. SHE (3)

Pb^2+^_(aq)_ + 2 e^−^ → Pb_(s)_    E° = −0.13 V vs. SHE (4)

Important research questions are needed to guide the scope of this study. The following are relevant research questions that deals with the objectives of the study:

1. Which MEC system performs efficiently in the removal of Fe^2+^ and Pb^2+^?
2. What are the mechanisms of heavy metal removal in these two MECs?
3. Does the use of MDC as a power source to the MEC perform more efficiently than the use of other power sources?
4. What are the mechanisms of ammonium ions’ transport from the middle chamber of the MDC?
5. Does the type of coupled MEC affect desalination in the MDC?
6. Do pH and conductivity affect the performance of the MDC–MEC coupled system?

## 2. Materials and Methods

### 2.1. MDC Construction

The cubic plexiglass MDC reactor was made up of anode (432 mL), desalination (144 mL), and cathode chambers (288 mL). Carbon electrodes with the projected surface area of 80 cm^2^ (16 × 5) were used as anode and cathode with carbon used as electron collectors. The electrodes were pre-treated with H_2_SO_4_ overnight and heated for an hour to activate the surfaces. The electrodes were connected to titanium wires that served as links between the electrodes and the lead connectors. The reactor was connected to a decadence resistor box at a resistance of 80 ohms to determine its performance. An anion exchange membrane (AEM, AMI-7001, Membranes International Inc., Ringwood, NJ, USA) was used to separate the anode chamber from the middle chamber and a cation exchange membrane (CEM, CMI-7000, Membranes International Inc., Ringwood, NJ, USA) was used to separate the middle chamber from the cathode chamber. The exchange membranes were pre-treated with NaCl (15 g/L) for at least 24 h and then rinsed with deionized water. A Ag/AgCl reference electrode was directly connected to the anode chamber to determine the anode potential.

### 2.2. MEC Construction

The cubic plexiglass dual-chambered MEC (DCMEC) reactor was made up of anode (162 mL) and cathode chambers (108 mL). The cubic plexiglass single-chambered MEC (SCMEC) reactor had a total working volume of 270 mL. The anode and cathode were carbon electrodes with a projected surface area of 17.25 cm^2^ (6.9 × 2.5) with the anode having carbon used as electron collectors. The electrodes were also pre-treated with H_2_SO_4_ overnight and heated for an hour to activate the surfaces. The electrodes were connected to titanium wires that served as links between the electrodes and the lead connectors. The reactor was connected to an external power supply (0.4–1.0 V) to determine its performance. The cathode was connected to the negative terminal of the power supply, while the anode was connected to the positive terminal through a 10 Ω resistor (a decadence box 0.1–9000 Ω). A cation exchange membrane (CEM, CMI-7000, Membranes International Inc., Ringwood, NJ, USA) was used to separate the anode chamber from the cathode chamber in the dual-chamber MEC. The exchange membrane was pre-treated with NaCl (15 g/L) for at least 24 h and then rinsed with deionized water.

### 2.3. Medium

The microbial consortium was cultured in hermetic containers for more than a week. The culture was comprised of wastewater from the Changchun Tianjia Sewage Treatment Plant (STP) in Changchun, China, and synthetic wastewater with glucose was the carbon source (50% *v*/*v*). Nitrogen gas was pumped into the hermetic container for about 5 min before and after they were filled with the culture. After several days, the 250 mL air-tight autoclaved conical flask was filled with 200 mL of the culture and 0.05 g of glucose was injected into it daily to enable the growth of the microorganisms under conducive anaerobic conditions. The temperature was maintained between 25 °C and 28 °C. This process was similar in both phases.

The MDC anolyte was prepared with the following composition: glucose 2.0 g/L; NH_4_Cl 0.05 g/L; NaHCO_3_ 0.05 g/L; NaCl 0.15 g/L; MgSO_4_·7H_2_O 0.01 g/L; CaCl_2_ 0.006 g/L; K_2_HPO_4_ 10.713 g/L; KH_2_PO_4_ 5.24 g/L; and 1 mL of trace elements [31] (Liaocheng Yuanze Chemical Co. Ltd., Liaocheng, China) in 1 L of deionized water. The desalination chamber contained 5 g/L of NH_4_Cl. Two (2) different catholytes, namely ferricyanide (K_3_[Fe (CN)_6_])-MDCF and 100 mM of potassium phosphate buffer-MDCP100 were used to determine the power generation and ammonium removal efficiency of the MDC.

The MEC anolyte was prepared with the following composition: sodium acetate 4.0 g/L; NH_4_Cl 0.05 g/L; NaHCO_3_ 0.05 g/L; NaCl 0.15 g/L; MgSO_4_·7H_2_O 0.01 g/L; CaCl_2_ 0.006 g/L; K_2_HPO_4_ 10.713 g/L; KH_2_PO_4_ 5.24 g/L; FeCl_2_·4H_2_O 0.067 g/L (0.05 g of Fe); and PbCl_2_ 0.178 g/L (0.05 g of Pb) in 1L of deionized water. The dual-chamber MEC anolyte (DCMEC) catholyte was comprised of 100 mM of potassium phosphate buffer; FeCl_2_·4H_2_O 0.067 g/L (0.05 g of Fe); and PbCl_2_ 0.178 g/L (0.05 g of Pb) in 1L of deionized water.

The anode chambers of both the MDC and MEC reactors were inoculated (50% *v*/*v*) with the cultured microbial consortium.

### 2.4. MDC–MEC Operation

**Figure 3:**
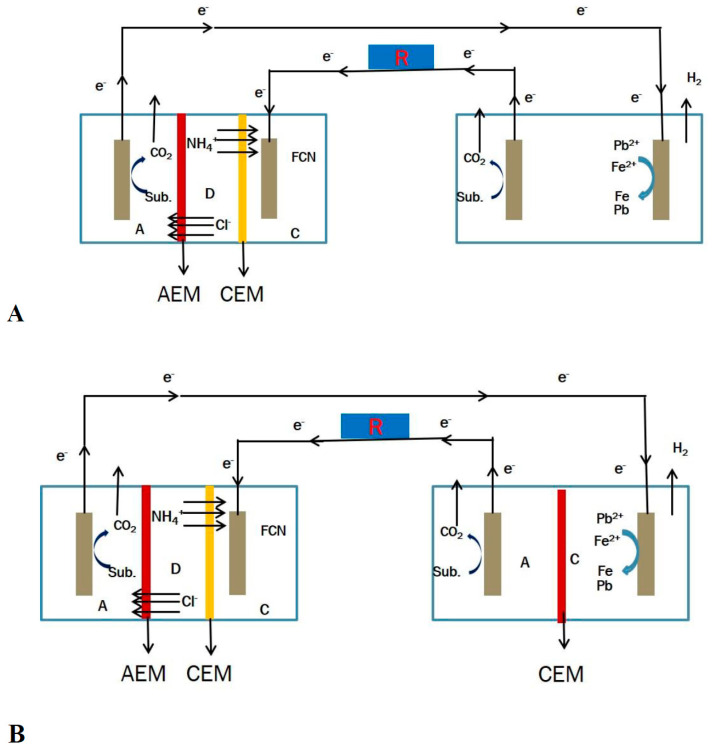
(**A**) Schematic diagram of the MDC–SCMEC coupled system. (**B**) Schematic diagram of the MDC–DCMEC coupled system. Note: A, anode chamber; C, cathode chamber; D, desalination chamber; AEM, anode exchange membrane; CEM, cathode exchange membrane; and R, external resistance.

The MEC was coupled with the MDC in series to enable the supply of power from the MDC to the MEC. The cathode of the MEC was connected to the anode of the MDC, while the anode of the MEC was connected to the cathode of the MDC through a 10 Ω resistor (Figure 3). All solutions were recirculated in the MDC at a flow rate of 5.75 mL/min using a peristaltic pump. This flowrate was chosen to allow for adequate hydraulic retention time (75.13 min) of the substrate to be oxidized at the anode chamber. Two MEC systems, SCMEC and DCMEC, were used to determine the removal efficiency and removal mechanism of the heavy metals in the reactors within a 48-h fed-batch cycle. MDCF was used as the power source for the MEC systems during the 48-h duration. The anolytes and catholytes in the MEC system were recirculated at a flow rate of 4.5 mL/min from their respective reservoirs (500 mL). All experiments were done in duplicates.

### 2.5. Controls

**Figure 4:**
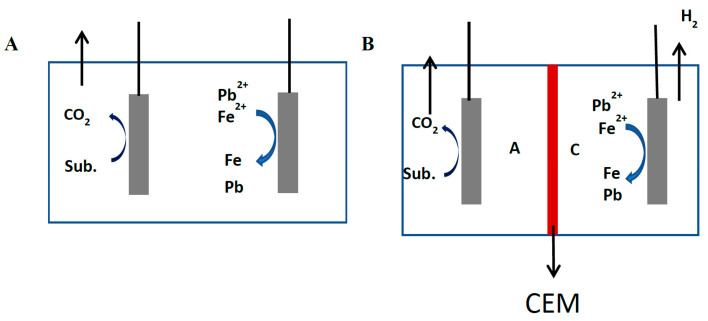
(**A**) A schematic diagram of the open circuit single-chamber MEC (Control 3). (**B**) A schematic diagram of the open circuit dual-chamber MEC (Control 4). Note: A, anode chamber; C, cathode chamber; D, desalination chamber; AEM, anode exchange membrane; CEM, cathode exchange membrane; R, external resistance; and Sub., substrate.

**Figure 5:**
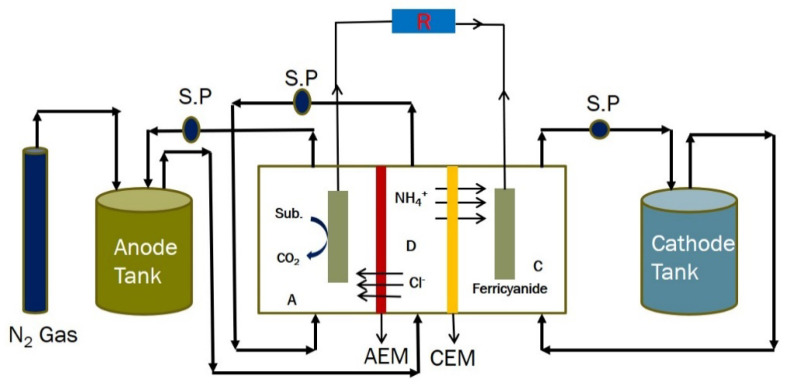
A schematic diagram of the MDC Control 2 with anolyte circulating through the desalination and anode chambers. Note: A, anode chamber; C, cathode chamber; D, desalination chamber; AEM, anode exchange membrane; CEM, cathode exchange membrane; R, external resistance; Sub., substrate; and S.P, sampling point.

Three (3) controls were set for the MDC–MEC systems. Open circuit systems were set as the controls for the SCMEC (Control 2) and DCMEC (Control 3) to determine the heavy metal removal efficiencies of the systems as seen in Figure 4. In the control for the MDC, the anolyte was circulated through both the desalination chamber and anode chambers with deionized water as the catholyte in the MDC, as shown in Figure 5. This MDC was coupled to an abiotic SMEC (the SCMEC was treated with ethanol to kill the microbes and render them abiotic) as Control 1. Control 1 was performed to determine the effect of a concentration gradient in the desalination and power generation in MDC and the coupling effect on the abiotic SCMEC system within 48 h. All experiments were done in duplicates. 

### 2.6. Analysis and Calculations

The voltage across the external resistance (10 Ω) of the MDC–MEC was continuously recorded every 1 min using a digital multimeter with a data logging system connected to a computer. The polarization curves were measured and calculated for the MDC and MEC systems with a series of external resistances (1 Ω–9000 Ω). The voltage over each external resistance was measured using a digital data-logging multimeter. The current was calculated according to I = V/R and the power was calculated according to P = I × V. The current and power densities were normalized by the anode surface areas of the MDC (80 cm^2^) and MEC (17.25 cm^2^). The pH of the solutions was measured using a portable digital pH meter. Conductivity was measured with a portable conductivity meter. The open-circuit potentials (OCPs) of the electrodes were measured using a data-logging potentiostat (NEV4). A Ag/AgCl electrode (+0.197 V vs. SHE) was used as a reference and the cathode or anode was the working electrode to test the OCPs of the electrodes. COD was measured using potassium dichromate titration. Iron (Fe^2+^) and lead (Pb^2+^) were measured using atomic absorption spectroscopy (AAS). Acetate, chlorine, phosphate, sodium, and potassium ions were measured using ion chromatography. Ammonium-nitrogen (NH_4_^+^–N) was measured with the DR/890 colorimeter (Hach Co., Ltd., Loveland, CO, USA) according to the manufacturer’s procedure. The salinity was estimated from the conductivity using a standard curve. Removal efficiency for COD, iron (Fe^2+^), and lead (Pb^2+^) was calculated as:

COD Removal Efficiency = ((COD_Initial_ − COD_Final_)/ COD_Initial_) × 100 (5)

where COD_Initial_ is initial concentration and COD_Final_ is final concentration. The removal rate for COD, iron (Fe^2+^), and lead (Pb^2+^) was calculated as:

COD Removal Rate = ∆COD/(V_A_ × d) (6)

where ∆C is the change of concentration (Kg), V_A_ is the volume of the anode, and d is the duration (days).

The desalination rate for NH_4_Cl was calculated as:

Desalination Rate = ∆COD/d(7)

where ∆C is the change of concentration (mg) and d is the duration (hours). MDC performance was determined by quantifying the number of coulombs produced from the substrate utilized, known as coulombic efficiency. For continuous flow through the system, coulombic efficiency (CE) is given as [32,33]:

CE = It/ (nFQ∆COD) × 100(8)

where 8 is a constant used for COD based on the molecular weight of O_2_ (32 g/mol) and the number of electrons exchanged per mole of oxygen (4 mol e–/mol O_2_). I represents the current measured from the MDC or MEC; F is Faraday’s constant (96485 C/mole-electrons); Q is the inlet flow rate of the MDC or MEC; and ∆COD is the change in COD over the fed-batch cycle.

## 3. Results and Discussion

### 3.1. Pb^2+^ and Fe^2+^ Removal in the MEC Systems within a 48-h Cycle

**Figure 6:**
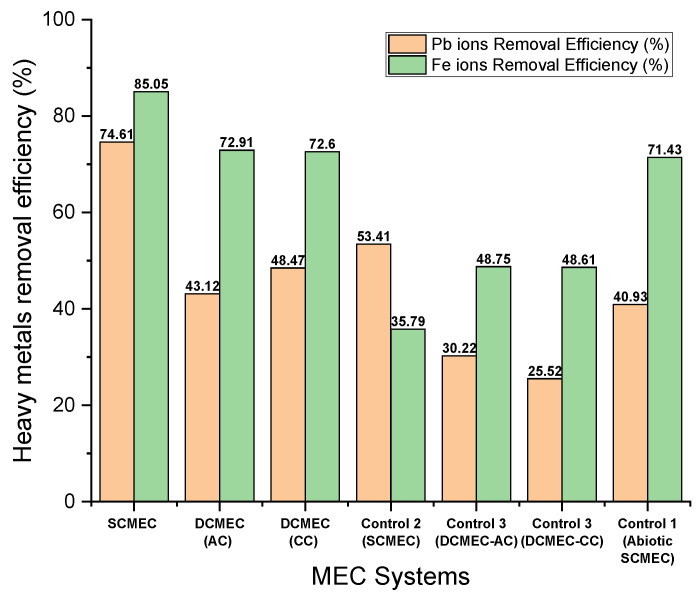
Heavy metals removal from the various MEC systems within a 48-h cycle.

**Figure 7:**
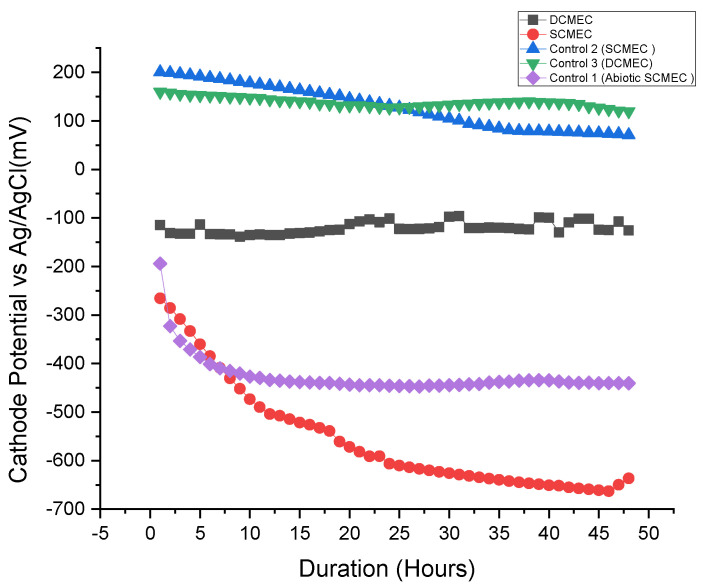
Cathode potentials of the various MEC systems within a 48-h cycle.

Two main removal mechanisms of Pb^2+^ and Fe^2+^ were seen in the MEC systems: (1) microbial biosorption and (2) cathodic reduction potential. Microbial biosorption involves the ability of microbes to accumulate heavy metals from wastewater through metabolically mediated (by the use of ATP) or spontaneous physicochemical pathways of uptake (not at the cost of ATP) [28,34,35,36]. The microbial biosorption was determined by placing Pb^2+^ and Fe^2+^ in the anode chamber of the DCMEC. The cathodic reduction potentials of Pb^2+^ and Fe^2+^ are −130 mV and −450 mV, respectively, as stated in Equations (3) and (4). Figure 6 describes the efficiency of various MEC systems in removing Pb^2+^ and Fe^2+^. The cathode potentials of the SCMEC, DCMEC, Control 1 (abiotic SCMEC), Control 2, and Control 3 during their 48 h of operation are shown in Figure 7. SCMEC had Pb^2+^ and Fe^2+^ removal efficiencies of 74.61% and 85.05%, respectively, with a corresponding cathode potential of −549 mV vs. Ag/AgCl, while DCMEC had Pb^2+^ and Fe^2+^ removal efficiencies of 48.47% and 72.91% respectively, in the cathode chamber with a corresponding cathode potential of −120.9 mV vs. Ag/AgCl. The SCMEC had the highest heavy metal removal efficiency within 48 h and higher than the DCMEC, Controls 1, 2, and 3 due to the simultaneous application of the two heavy metal removal mechanisms. The DCMEC had Pb^2+^ and Fe^2+^ removal efficiencies of 43.12% and 72.91%, respectively, in the anode chamber, which shows that combining the two mechanisms provides a higher removal efficiency than a single mechanism of heavy metal removal provides. Other research studies confirm the high heavy metal removal in the SCMEC [37,38]. The required electrode potentials to reduce Pb^2+^ (−0.13 to −0.267 V vs. SHE) and Fe^2+^ (−0.45 to −0.51 V vs. SHE) were more negative than the anode potentials under open circuit of the DCMEC (−0.212 V vs. Ag/AgCl) and SCMEC (−0.079 V vs. Ag/AgCl). In open-circuit operations, the exoelectrogens are restricted in transferring electrons to the anode. Therefore, exoelectrogens gain energy by transferring their electrons to heavy metals (Fe^2+^ and Pb^2+^). The restriction of electron transfer to the anode must have enabled Pb^2+^ and Fe^2+^ to be electron acceptors at the anode during the open circuit experiment, resulting in the Pb^2+^ and Fe^2+^ reduction in the open circuits [37]. Biosorption also plays a major role in Pb^2+^ and Fe^2+^ removal in the open circuits. Control 1, 2, and 3 were performed to determine the efficiency of each mechanism. Control 1 (abiotic SCMEC) had the highest Pb^2+^ and Fe^2+^ removal efficiency with a corresponding cathode potential of −425.7 mV vs. Ag/AgCl. This process showed that the cathodic reduction potential in heavy metals removal is more efficient than that of microbial biosorption as metal affinity, saturation of active sites of metal-binding ligands, pH, temperature, inhibitions from by-products, and even the types of heavy metals may affect microbial biosorption [34,35,36]. The general range of pH for metal uptake is between 2.5 and 6. Above this limit, the metal uptake ability of bio-sorbent microbes gets compromised [34,35,36].

### 3.2. Effect of COD and Desalination on Electricity Generation in the Different MECs

**Figure 8:**
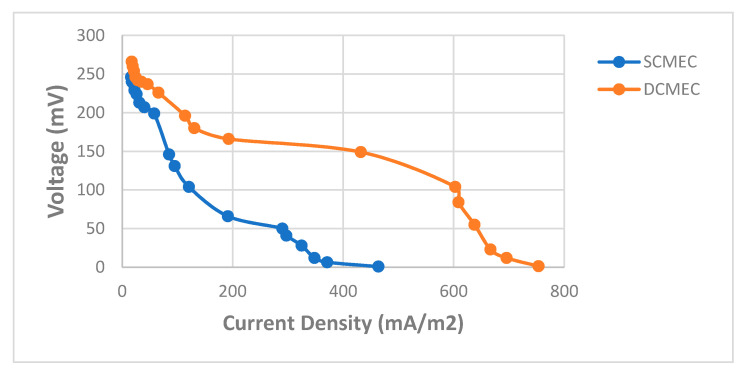
Polarization curves of the SCMEC and DCMEC.

**Figure 9:**
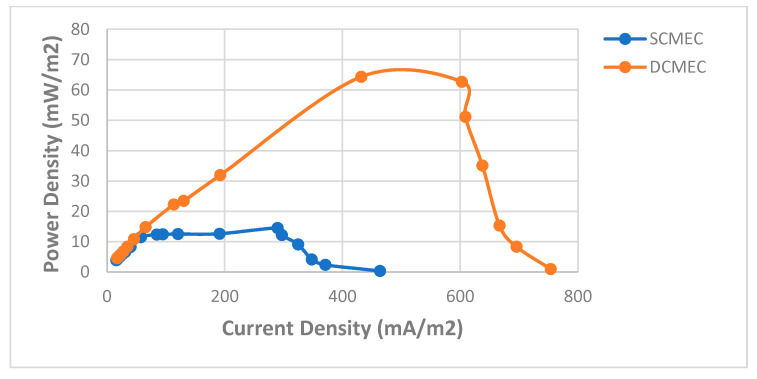
Power curves of the SCMEC and DCMEC.

**Figure 10:**
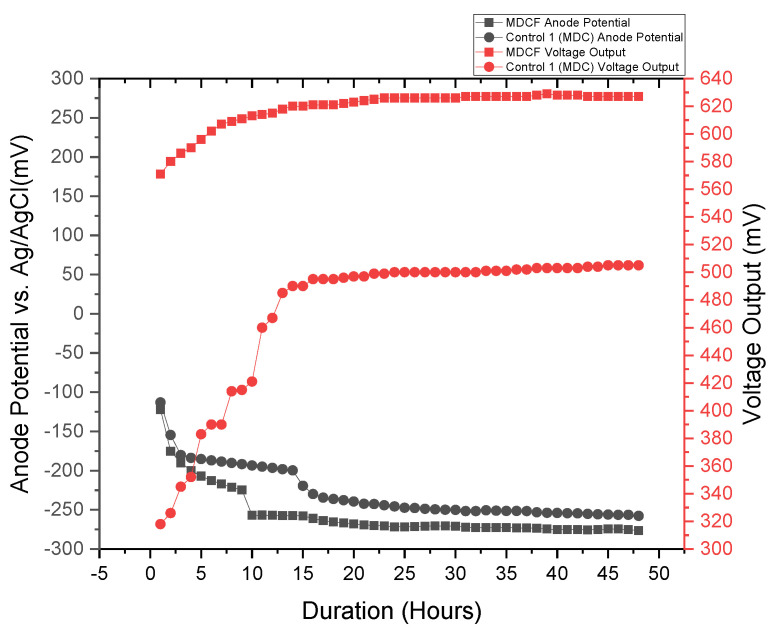
The anode potential and voltage output of the MDCF and Control 1 as a power source within a 48-h cycle.

COD removal in the bioreactors is essential for the desalination process. Glucose and acetate are simple carbon sources that are easily degraded by the exoelectrogens to gain energy. The use of acetate in MEC affects the microbial diversity in the biofilm. It has been reported that Geobacter and Pelobacter species are dominant when acetate is a carbon source in the MEC [39]. The current generation is greatly influenced by the oxidation of the carbon sources in the bioreactors. The SCMEC had a COD removal efficiency of 30.54%, while DCMEC had a COD removal efficiency of 27.88% within 48 h (Table 1.). At the end of the 48-h cycle, the exoelectrogens had access to the substrate that can be oxidized for energy gain and current generation which will lead to the energy efficiency of the bioreactors in removing the subsequent addition of Pb^2+^ and Fe^2+^ in the wastewater. The DCMEC had a maximum current and power density of 33.599 mAm^−2^ and 49.559 mWm^−2^, respectively, which were significantly higher than that of the SCMEC (17.995 mAm^−2^ and 14.216 mWm^−2^) at 10 Ω within a 48-h cycle (Table 1, Supplementary Figure S1). The CEM in the DCMEC creates an ionic concentration gradient that greatly influences the flow of the current in the bioreactor. The anode potentials of the SCMEC and DCMEC were −224.3 mV and −256.3 mV vs. Ag/AgCl, respectively, which corresponded with their power densities. The maximum current densities in the MECs were obtained at the highest anode potentials, which is consistent with other research studies [40]. Previous studies have assessed the impact of anode potential on the development of the bio-electroactive biofilms in different MEC systems (including single chamber and dual chamber MECs) [40]. The change in anode potentials leads to the diversification of the microbial population, which helps in substrate oxidation and electron production and transfer [40,41,42]. The thermodynamic energy of the electron donor and receptor, and the efficiency of the electron transport chain, influence the energy needed by exoelectrogenic bacteria (obtained through electron transfer from the microbes to the anode [17]). Thus, the anode potential significantly affects the distribution and growth of exoelectrogens. The SCMEC and DCMEC had low coulombic efficiencies of 1.21% and 2.7%, respectively. The low coulombic efficiency in both MECs might be due to external resistance; microbial cell biomass production; the presence of other electron-accepting species; and other competitive electron-consuming reactions such as methane production [24]. Methane production is a usual occurrence in MEC [43]. The presence of hydrogenotrophic methanogens consuming H_2_ at the cathode in the SCMEC might also influence the low CE [39]. In the DCMEC, the exoelectrogenic bacteria growing in the anode chamber were completely separated from the cathode, resulting in more than double the CE as compared to that of the SCMEC [24,43]. The current density and CE of the MECs affected the removal of ammonium in the MDCF. From the polarization curves, the DCMEC had a higher current density of 753.62 mAm^−2^ than that of SCMEC with a current density of 463.77 mAm^−2^ at a resistance of 1 Ω (Figure 8). Ohmic and concentration losses were dominant in the SCMEC, while concentration losses were dominant in the DCMEC [32]. From the power curves, the DCMEC had a higher power density of 64.35 mWm^−2^ at 200 Ω than that of SCMEC with a power density of 14.49 mWm^−2^ at 100 Ω (Figure 9). The high current density and CE of the DCMEC resulted in 29.09% of desalination in the MDCF as compared to the 18.34% of desalination due to the low current density and CE of the SCMEC (Supplementary Figure S2). Control 2 and 3 had a COD removal efficiency of 16.91% and 24.05%, respectively, at the anode potentials of −79.7 mV and −211.9 mV vs. Ag/AgCl (Table 1). Though Control 2 and 3 were open circuits, the presence of CEM in Control 4 enabled the transfer of protons and cations from the anode chamber to the cathode chamber, which caused a potential difference between the two chambers. This potential difference enabled the anode to accept electrons from the exoelectrogenic bacteria, which caused it to have a high anode potential as compared to Control 2 (open circuit SCMEC). The exoelectrogens in Control 3 were then able to oxidize more substrate than that of Control 2. The MDCF and Control 1 (MDC) had an average COD removal efficiency of 73.28% (Supplementary Figure S2) and 68.28%, respectively, with corresponding maximum voltage outputs of 627 mV and 505 mV (Table 2). The voltage output from the MDC system within 48 h was higher than that of other related systems in literature as shown in Table 2. The increase in voltage output was due to the high COD removal efficiency, conductivity, and electron accessibility of the MDC system. The MDCF and Control 1 (MDC) had stable anode potentials at −276.6 mV and −257.7 mV vs. Ag/AgCl during the 48-h cycle, which corresponded with the respective voltage outputs (Figure 10). The maximum voltage outputs were obtained at the highest anode potentials. Though the difference in the anode potentials was not very significant, a very significant difference is seen in the voltage output produced. Two major factors influenced the low voltage output in Control 1 (MDC) as compared to the voltage output in MDCF. The recirculation of the same wastewater in both the anode and middle chambers of the MDC in Control 2 affected the concentration gradient between the two chambers, as there was little to no ion exchange between the two chambers. Thus, this factor greatly influenced the voltage output. The second major factor involved the fact that there was no current flow between the anode of the abiotic SCMEC and the cathode of the MDC in Control 1. The catholyte of the MDC in Control 1 was deionized water, which is a low electron acceptor as compared to ferricyanide as the catholyte. No electrons were produced in the abiotic SMEC as it had in the exoelectrogenic bacteria to oxidize the substrate. Though the anode of the MDC in Control 1 supplied electrons to the cathode of the abiotic SCMEC, the cathode (without receiving electrons) affected the potential difference in the MDC, which greatly influenced the voltage output. Thus, there is a synergic influence of the MDC and MEC bioreactors in the optimum performance of the coupled system.

### 3.3. Effect of pH and Conductivity within a 48-h Cycle

Another key indicator of the performance of the MDC–MEC coupled system is the pH and conductivities of the electrolytes. The performance of these indicators reflects on other parameters such as desalination, COD removal, the current generation, and heavy metals removal. Table 3 shows the initial and final pH and conductivities of the SCMEC, DCMEC, their controls, and the middle chamber of the coupled MDC within 48 h.

The low pH of the anolyte in the bioreactor can inhibit the activity of the exoelectrogens [44,45]. Recirculation and buffering of the electrolytes are used to stabilize the pH [44]. The anolytes were buffered and all the solutions were recirculated in the MDC–MEC coupled system. In previous studies, the pH dropped fast in the anode chamber after the first day for both experimental and control MFC units, which dropped the conductivity and power generation of the MFC [22]. Very high or low conductivities of the electrolytes can negatively affect COD removal, the current generation, and desalination. There was no significant difference in the initial and final pH of the electrolytes in the MECs. An average pH of 7.05 ± 0.5 was obtained during the operation of the coupled system. The pH of the middle chamber in the MDCF increased from a slightly acidic pH of 5.83 to a neutral pH of 7.08. This pH was conducive for the growth and catabolic activity of the exoelectrogenic bacteria and for the transfer of ions in the coupled system, which indicated the efficiency of the whole system. The conductivities of the anolytes and catholytes in the MECs slightly increased after the 48-h cycle. The increase in the anolyte conductivity may be due to the release of microbial by-products such as CO_2_. In the DCMEC, a slight increase in the catholyte conductivity may be a transfer of cations and protons from the anode chamber. The conductivity of the middle chamber in the MDCF average decreased from 9.37 mScm^−1^ to 8 mScm^−1^ after the 48-h cycle.

## 4. Conclusions

The recirculatory MDC–MEC coupled system is a novel technology that produces electric power from nitrogen removal and desalination, and supports metals removal from wastewater through eco-friendly processes. Unlike conventional methods such as chemical precipitation, reverse osmosis, and electrochemical treatment, rMDC–MEC is energy efficient by simultaneously using microbial biosorption and cathodic reduction for heavy metals removal in the MEC, as well as removing ammonium ions in the middle chamber of the MDC by using the MDC as the power source. The SCMEC had a higher Pb^2+^ (74.61%) and Fe^2+^ (85.05%) removal efficiency during the 48-h cycle than the DCMEC due to the simultaneous use of microbial biosorption and the cathodic reduction potential. The DCMEC had a higher current and power density of 33.599 mAm^−2^ and 49.559 mWm^−2^, respectively, and a CE of 2.7%, which influenced higher desalination in the MDCF than in the SCMEC within the 48-h cycle. The MDCF produced a higher voltage than Control 2 (MDC) did as a power source to the MECs. Stable electrolytes’ pH and conductivities provided a conducive operation of the coupled system. Although ferricyanide produces a very high current density and drives high desalination, it is not sustainable and cannot be reused after an experiment. A sustainable and efficient catholyte, such as the use of algae, fungi, or air-cathode, in the rMDC–MEC needs to be investigated. The use of nanocomposite materials in both the MEC and MDC will greatly enhance the heavy metals and ammonium removal efficiencies and reduce biofouling in the coupled system during a long-term operation [20]. This study lays a solid background for the type of MDC–MEC coupled systems needed for industrial scale-up. The choice of the coupled system will be dependent on the key parameters (such as desalination, power generation, heavy metals removal, and hydrogen production) that will be investigated. The volume ratio of the COD and NH_4_Cl removal in the anode and middle chambers of the MDC for balanced COD/ammonium ions’ removal is a key driving force to the scale-up of the coupled system.

## Figures and Tables

**Table 1:** Efficiency of the various MEC systems within a 48-h cycle.

Systems	COD Removal Efficiency (%)	Cathode Potential (mV)	Anode Potential (mV)	Maximum Current Density at 10 Ω (mA/m^2^)	Maximum Power Density at 10 Ω (mW/m^2^)	Desalination in MDC (NH_4_Cl) (%)	CE (%)
SCMEC	30.54	−549	−224.3	17.995	14.216	18.34	1.21
DCMEC	29.45	−120.9	−256.3	33.599	49.559	29.09	2.7
Control 1 (Abiotic SCMEC)	-	−425.7	215.7	-	-	-	-
Control 2 (SCMEC)	16.91	129.5	−79.7	-	-	-	-
Control 3 (DCMEC)	24.05	137.5	−211.9	-	-	-	-

**Table 2:** Efficiency of the MDCF and Control 1 (MDC) as a power source within a 48-h cycle in comparison to other related systems.

Systems	COD Removal Efficiency (%) within 48 h	Anode Potential (mV)	Maximum Voltage Output (mV)	Reference
MDCF	73.28	−276.6	627	Experiment
Control 1 (MDC)	68.28	−257.7	505	Experiment
MDC-MEC	62.9	-	370	[23]
MFC-MEC	-	-	504	[24]
MFC-MEC	-	-	400	[22]

**Table 3:** pH and conductivity of the SCMEC, DCMEC, and NH_4_Cl (MDC) within a 48-h cycle.

Systems	Initial pH	Final pH	Initial Conductivity (mS/cm)	Final Conductivity (mS/cm)
SCMEC	7.52	7.47	15.62	16.35
DCMEC (AC)	7.04	7.02	16.09	17.02
DCMEC (CC)	7.08	6.97	13.09	13.41
MDC (NH4Cl)	5.83	7.08	9.37	8
Abiotic SCMEC Control 1	7.47	7.08	14.82	14.12
SCMEC Control 2	7.38	7.26	14.94	15.8
DCMEC (AC) Control 3	7.48	7.39	14.7	14.86
DCMEC (CC) Control 3	7.61	7.17	12.01	12.24

## Data Availability

Not applicable.

## Supplementary Materials

The following are available online at https://www.mdpi.com/article/10.3390/membranes11090661/s1, Figure S1: Current and Power Densities performance of the SCMEC and DCMEC at 10 Ω within a 48-h cycle; Figure S2: COD removal efficiencies and Desalination in MDC over 8 fed-batches as a power source to the MEC systems.
