# Supplementary material for: Comparative Studies of Recirculatory Microbial Desalination Cell–Microbial Electrolysis Cell Coupled Systems
Source: Membranes (Basel). 2021 Aug 27;11(9):661. doi: 10.3390/membranes11090661 (PMC8470946; doi:10.3390/membranes11090661)
Supplement: Supplementary file 1 [file membranes-11-00661-s001.zip › membranes-1341610-supplementary.pdf]

SUPPLEMENTARY

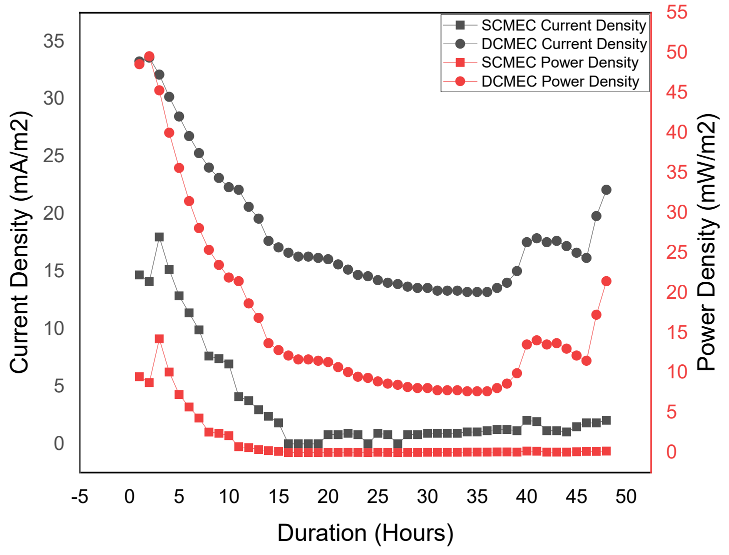

Figure S1. Current and Power Densities performance of the SCMEC and DCMEC at 10Ω within a 48-hour cycle.

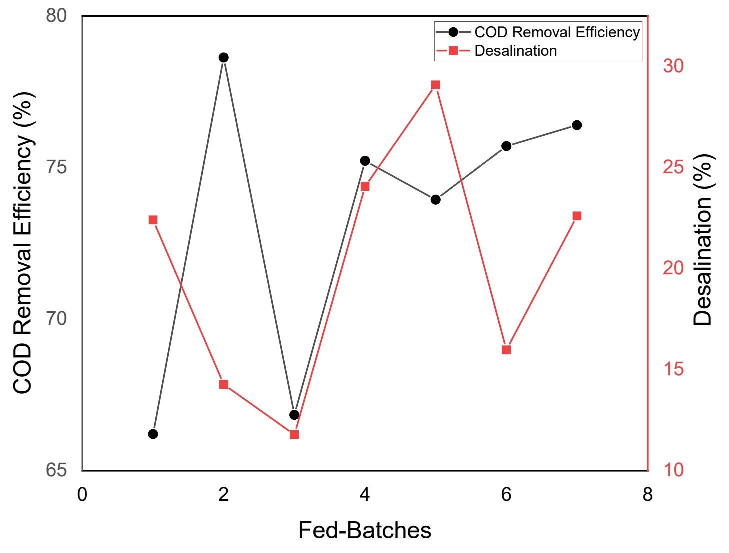

Figure S2. COD removal efficiencies and Desalination in MDC over 8 fed-batches as a power source to the MEC systems.
